# Use of ICD-10 diagnosis codes to identify seropositive and seronegative rheumatoid arthritis when lab results are not available

**DOI:** 10.1186/s13075-020-02310-z

**Published:** 2020-10-15

**Authors:** Jeffrey R. Curtis, Fenglong Xie, Hong Zhou, David Salchert, Huifeng Yun

**Affiliations:** 1 Division of Clinical Immunology & Rheumatology, University of Alabama at Birmingham, Birmingham, AL USA; 2 Department of Epidemiology, University of Alabama at Birmingham, Birmingham, AL USA; 3 Department of Computer Science, University of Alabama at Birmingham, Birmingham, AL USA

**Keywords:** Rheumatoid arthritis, Electronic health records, Algorithm, Claims data, Validity, ICD-10

## Abstract

**Background:**

Rheumatoid factor (RF) and anti-cyclic citrullinated peptide (CCP) antibody tests are often measured at the time of rheumatoid arthritis (RA) diagnosis but may not be repeated and therefore not available in electronic health record (EHR) data; lab test results are unavailable in most administrative claims databases. ICD10 coding allows discrimination between rheumatoid factor positive (M05) (“seropositive”) and seronegative (M06) patients, but the validity of these codes has not been examined.

**Methods:**

Using the ACR’s Rheumatology Informatics System for Effectiveness (RISE) EHR-based registry and U.S. MarketScan data where some patients have lab test results, we assembled two cohorts. Seropositive RA was defined having a M05 diagnosis code on the second rheumatologist encounter, M06 similarly identified seronegative RA, and RF and anti-CCP lab test results were the gold standard. We calculated sensitivity (Se) and positive predicted value (PPV) of the M05/M06 diagnosis codes.

**Results:**

We identified 43,581 eligible RA patients (RISE) and 1185 (MarketScan) with RF or anti-CCP lab test results available. Using M05 as the proxy for seropositive RA, sensitivity = 0.76, PPV = 0.82 in RISE, and Se = 0.73, PPV = 0.84 in MarketScan. Results for M06 as a proxy for seronegative RA were comparable in RISE, albeit somewhat lower in MarketScan. Over 3 consecutive visits, approximately 90% of RA patients were coded consistently using either M05 or M06 at each visit.

**Conclusion:**

Under ICD10, M05 and M06 diagnosis codes are reasonable proxies to identify seropositive and seronegative RA with high sensitivity and positive predictive values if lab test results are not available.

## Background

Large electronic databases are increasingly used in healthcare research to generate real-world evidence [1]. Registries derived from large-scale electronic health record (EHR) systems and administrative databases from large health plans are an important component of this data infrastructure. However, like all data sources, they are typically incomplete in some aspects. For example, administrative claims data often lack lab results. Moreover, both claims and EHR data are subject to left censoring, in which patients have their data represented only from the time that they are enrolled in the health plan (for claims data sources) or receiving care from their physician from whom the EHR data is available [2]. Nothing is known about the patient prior to that time. Left censoring is particularly important for lab tests and diagnostic studies that are typically performed once at the time of diagnosis, given that these diagnostic tests are usually not repeated since they are not expected to change over time in patients with an established diagnosis.

The shift in the USA from the International Classification of Diseases, 9th edition (ICD-9), to the 10th edition (ICD-10) that occurred in October of 2015 greatly increased the number of diagnostic codes available to classify patient’s medical condition. The corpus of approximately 13,000 ICD-9 codes was expanded by more than five-fold to more than 69,000 codes. Some of these codes were used to confer additional specificity in diagnoses, disease subtypes, or to denote complications (e.g., diabetes) [3]. Some codes allowed for indication of body site with laterality (e.g., fracture of the left femur), and some provided information about results of lab tests [4]. For example, in rheumatology, the previous diagnosis code most commonly used for rheumatoid arthritis (ICD-9 714.0) that provided no information about lab testing was replaced with a family of codes to describe patients as being positive for the rheumatoid factor (RF) lab test, e.g., M05.0 “rheumatoid arthritis with (positive) rheumatoid factor” or negative for RF (e.g., M06.0) “rheumatoid arthritis without rheumatoid factor”. Given that RF is an important biomarker of prognostic significance for clinical and radiographic outcomes for RA patients [5–7], the availability of these ICD-10 codes is potentially valuable for clinical research when using data sources where the actual lab results are not available.

However, the validity of these codes to accurately identify the lab tests that they seemingly proxy is unknown, as is the use of these codes to reflect anti-citrullinated protein antibody (ACPA) lab test results. We examined coding behaviors and the validity of the M05 and M06 ICD-10 codes compared to the gold standard of lab test results in both a large national U.S. rheumatology registry, the American College of Rheumatology’s (ACR) Rheumatology Informatics System for Effectiveness (RISE), and the large U.S. administrative database, MarketScan. We hypothesized that the M05 and M06 codes would accurately proxy for RF and ACPA lab test results and that coding for any given patient would be consistent over time, as would be expected for these two lab tests whose values do not typically fluctuate over time.

## Methods

We created two separate cohorts of rheumatoid arthritis (RA) patients using October 1, 2015–December 31, 2017, RISE and MarketScan data. RA patients were required to have two or more rheumatologist’s diagnosis codes for RA (M05.* or M06.*, ignoring M06.1 and M06.4), assigned on an office visit encounter and occurring between 7 and 365 days of one another [8]. They were also required to have a prescription or an administration of a conventional synthetic, targeted synthetic, or biologic disease-modifying anti-rheumatoid drug (DMARD). The index date was defined as the date that the patient met both the diagnosis code and DMARD criteria.

### Validity of diagnosis codes to proxy for seropositive and seronegative RA

To be included in the analysis for assessing the validity of M05.* as a proxy for seropositivity and M06.* as a proxy for seronegativity, RA patients were required to had at least one lab test with valid numeric test results or dichotomous (Yes/No) results for rheumatoid factor (RF) or anti-cyclic citrullinated peptide (anti-CCP). With the expectation that RF and anti-CCP lab test results largely are time invariant, we examined all lab results assessed at any time using all available data up until the 2nd M05/M06 diagnosis code. For RF, numeric results ≥ 14 IU/ml were defined as positive based on the common upper lab limit of normal, and for anti-CCP antibody tests, ≥ 20 was defined as positive. The lab test was used as gold standard, and the upper limit of lab normal was confirmed in each data source. Additional analyses were conducted that defined high-positive lab values as those more than three times the upper limit of normal (RF > 42, CCP > 60), and those patients with low-positive results were excluded. Because RF and anti-CCP lab test results initially might be negative in early RA and subsequently become positive on repeat testing, if a patient had more than one RF or anti-CCP lab test result, it was classified as positive if any of them were positive, up to the date of the 2nd M05/M06 diagnosis code.

The M05 or M06 diagnosis assigned as the 2nd ICD 10 diagnosis code was the main independent variable. Moreover, we required that there be a gap of > 30 days between the lab test and this diagnosis code, in as much as the lab test result must be known in order for the diagnosis to have been coded accurately. To explore the importance of this requirement, we then evaluated the agreement between a diagnosis code for M05 and seropositivity according to the interval of time between the lab test result and the diagnosis code.

### Consistency of RA coding over time and by individual rheumatologists

To evaluate the consistency of RA coding over time in patients who continue to receive care from a rheumatologist, we conducted separate analysis requiring patients to have at least three rheumatologist visits with a RA diagnosis code. Having RF or ACPA lab results available were not required.

To be included in the analysis for assessing the variability within rheumatologist practices in assigning M05 and M06 codes to his/her RA patients, individual rheumatologists were identified using National Provider Identifier (NPI) numbers or other unique identifiers and were required to see at least 10 RA patients. Within the practice of each rheumatologist, the proportion of ever use of an M05 or M06 diagnosis code was calculated as the number of RA patients ever assigned an M05 or M06 diagnosis code divided by total number of RA patients treated by that rheumatologist. The purpose of this analysis was to evaluate whether some rheumatologists might always, or never, use the M05 or M06 diagnosis code for their patients, suggesting that coding practices did not follow the actual seropositive or seronegative status.

### Statistical analysis

Descriptive statistics were used to characterize both RA cohorts in the RISE and MarketScan data, comparing those with RF and/or anti-CCP lab test results available versus to those where it was not available. The Charlson Comorbidity Index was used to classify comorbidities [9]. Standardized mean differences (SMDs) were used to compare characteristics, with SMD > 0.10 used to identify potentially important differences. Sensitivity (Se), positive predicted value (PPV), and agreement (kappa) with 95% confidence interval were calculated for the occurrence of M05 and M06 codes compared to various lab-based gold standards. Specificity was not reported separately because the results of the M05 and M06 analyses are inter-related; a “M05 negative” diagnosis code is synonymous with a “M06 positive” diagnosis code. Therefore, for the comparison to single lab test results, the specificity of M05 diagnosis codes is the same as the sensitivity of M06 diagnosis codes to classify a negative test.

A Sankey plot was drawn to show switching patterns in the use of M05 and M06 codes for the first three rheumatology visits in the observation period. Additional analysis also was conducted to examine whether rheumatologists always or never used M05 or M06 diagnosis codes for all their RA patients. Rheumatologists were grouped as to whether they used the M05 diagnosis code for 0%, between 0 and 25% of patients, 50–75%, 75 to < 100%, or all of them (100%). The use of the data was governed by data use agreements, and the analysis was approved by the university institutional review board. SAS 9.4 was used to carry out all analyses.

## Results

The attrition table for cohort selection in both the RISE and MarketScan data is shown in Additional file 1. A total of and 134,406 (RISE) and 78,787 (MarketScan) patients were eligible for analysis. The majority (> 85%) of RISE patients who were tested had lab results for RF and/or anti-CCP antibody available, whereas the lab results were available only for a minority (7%) of tested patients within the MarketScan data. Characteristics of patients eligible for analysis according to whether they were tested or now are shown in Table 1. In general, there were very few differences (based on SMD > 0.10) within each dataset according to testing status, with only a few exceptions. In RISE, patients tested and with results available were slightly younger (mean age 60.7 years versus 62.4 years in those not tested). Glucocorticoid use was also more common in those tested (45.3% vs. 37.1%). In MarketScan, tested RA patients were younger and had somewhat higher comorbidity scores and a higher prevalence of specific comorbidities (e.g., diabetes) and were less likely use to use biologics but more likely to use NSAIDs. Overall, 57% of RA patients in RISE, and 69% of those in MarketScan, were seropositive for RA and/or anti-CCP antibody, among those tested where results were available.

**Table 1:** Baseline* characteristics of RA patients in RISE EHR and MarketScan data according to the testing status for rheumatoid factor and anti-CCP antibody

	RISE EHR (*n* = 134,406)				MarketScan (*n* = 78,787)			
	Not tested	Tested, without results**	Tested, with results**	SMD	Not tested	Tested, without results**	Tested, with results **	SMD
N	72,432	8710	53,264		46,676	29,925	2186	
Age	62.5 (13.5)	61.3 (14.0)	60.7 (14.0)	0.0877	56.82 (12.70)	52.13 (11.54)	54.04 (12.19)	0.2572
Female	55,930 (77.2)	6758 (77.6)	41,365 (77.7)	0.0225	36,376 (77.9)	23,485 (78.5)	1735 (79.4)	0.0234
Charlson Comorbidity Index				0.0363				0.0973
1–2	68,170 (94.1)	8118 (93.2)	46,948 (93.2)		31,615 (67.7)	20,410 (68.2)	1366 (62.5)	
3–4	3938 (5.4)	561 (6.4)	3334 (93.2)		9351 (20.0)	6330 (21.2)	517 (23.7)	
≥ 5	324 (0.4)	31 (0.4)	282 (0.5)		5710 (12.2)	3185 (10.6)	303 (13.9)	
Comorbidities***								
Cerebrovascular disease	556 (0.8)	54 (0.6)	375 (0.7)	0.0119	5081 (10.9)	2847 (9.5)	271 (12.4)	0.0616
Congestive heart failure	364 (0.5)	47 (0.5)	258 (0.5)	0.0052	2951 (6.3)	1502 (5.0)	148 (6.8)	0.0496
Constructive pulmonary disease	2612 (3.6)	332 (3.8)	1856 (3.5)	0.0116	13,155 (28.2)	9010 (30.1)	638 (29.2)	0.0282
Diabetes without complication	3076 (4.2)	438 (5.0)	2961 (5.6)	0.0406	5777 (12.4)	4262 (14.2)	366 (16.7)	0.0827
Diabetes with complication	289 (0.4)	28 (0.3)	260 (0.5)	0.0175	2774 (5.9)	1643 (5.5)	158 (7.2)	0.0475
Malignancy	1302 (1.8)	173 (2.0)	954 (1.8)	0.0096	3837 (8.2)	2108 (7.0)	172 (7.9)	0.0295
Myocardial infarction	67 (0.1)	7 (0.1)	47 (0.1)	0.0028	1559 (3.3)	748 (2.5)	61 (2.8)	0.0333
Mild liver disease	957 (1.3)	163 (1.9)	1134 (2.1)	0.0415	4673 (10.0)	3762 (12.6)	327 (15.0)	0.1001
Peptic ulcer disease	345 (0.5)	47 (0.5)	284 (0.5)	0.0059	406 (0.9)	262 (0.9)	26 (1.2)	0.0211
Peripheral vascular disease	453 (0.6)	72 (0.8)	398 (0.7)	0.0158	1360 (2.9)	939 (3.1)	79 (3.6)	0.0263
Stroke	234 (0.3)	18 (0.2)	151 (0.3)	0.0151	1479 (3.2)	819 (2.7)	85 (3.9)	0.043
Medication use, %								
Methotrexate	39,177 (54.1)	4567 (52.4)	29,128 (54.7)	0.0301	26,185 (56.1)	17,789 (59.4)	1371 (62.7)	0.09
Other csDMARDS	28,950 (40.0)	3927 (45.1)	24,042 (45.1)	0.0698	19,894 (42.6)	14,915 (49.8)	996 (45.6)	0.0967
TNFi biologics	18,151 (25.1)	1961 (22.5)	11,443 (21.5)	0.0565	19,030 (40.8)	8204 (27.4)	525 (24.0)	0.2421
Non-TNFi biologics	6495 (9.0)	663 (7.6)	3662 (6.9)	0.0517	6593 (14.1)	2814 (9.4)	183 (8.4)	0.122
Targeted synthetic DMARDs	2694 (3.7)	342 (3.9)	1638 (3.1)	0.0309	2048 (4.4)	983 (3.3)	48 (2.2)	0.0824
NSAIDs	24,610 (34.0)	3273 (37.6)	20,631 (38.7)	0.0660	20,925 (44.8)	18,289 (61.1)	1332 (60.9)	0.2204
Opioids	14,177 (19.6)	1799 (20.7)	11,123 (20.9)	0.0217	21,849 (46.8)	16,795 (56.1)	1197 (54.8)	0.1247
Glucocorticoid use	26,889 (37.1)	3552 (40.8)	24,132 (45.3)	0.1111	34,034 (72.9)	25,335 (84.7)	1852 (84.7)	0.1947
Seropositive by RF and/or CCP, %	N/A	N/A	30,530 (57.3)		N/A	N/A	1511 (69.1)	
M05 diagnosis code (rather than M06), %	46,084 (63.6)	4753 (54.6)	29,245 (54.9)	0.1233	25,728 (55.1)	15,584 (52.1)	1226 (56.1)	0.0536

*SMD* standardized mean difference, SMDs > 0.10 are potentially important; *DMARD* disease-modifying anti-rheumatic drug; *TNFi* tumor necrosis factor inhibitor; *NSAID* non-steroidal anti-inflammatory drugs. Baseline refers to the date of the 2nd ICD-10 diagnosis code for RA

**Tested with results means that the patient was tested for either or both RF and anti-CCP antibody (e.g., based on billing claims for the relevant lab tests) and had a valid lab result available; tested without results means that they were tested, but results were not available in the dataset; ***as measured in the Charlson Comorbidity Index

Using RF positivity as the gold standard (Table 2), the sensitivity for seropositivity using any M05 diagnosis code was 0.82 (0.81–0.82) and the PPV was 0.81 (0.80–0.82) in RISE, and 0.73 (0.70–0.76) and 0.84 (0.81–0.87) in MarketScan. Using CCP as the gold standard, sensitivity was lower at 0.76 (0.75–0.76) and PPV was 0.68 (0.67–0.69) in RISE, and 0.64 (0.56–0.71) and 0.76 (0.68–0.83) in MarketScan. Combining (RF or CCP) as the gold standard, the sensitivity of the ever use of the M05 diagnosis code was 0.76 (0.75–0.76), PPV 0.82 (0.82–0.83) in RISE, and 0.73 (0.69–0.77) and 0.84 (0.81–0.87) in MarketScan. Requiring additional diagnosis codes, or examining the last code, minimally improved Se and PPV (not shown). The corresponding sensitivities and PPVs for the M06 diagnosis code to identify seronegative patients were comparably high in RISE. Both were approximately 80% for RF and slightly lower for anti-CCP. The parallel results for sensitivity and PPV in the MarketScan data for M06 coding were lower, albeit with much smaller sample size compared to RISE. They were numerically better once the low positive lab tests results were excluded (sensitivity = 0.69, 0.64–0.73; PPV = 0.71, 0.67–0.76).

**Table 2:** Sensitivity, positive predictive value, and agreement of M05 and M06 diagnosis codes in RA patients compared to various lab-based gold standards

		RISE EHR				MarketScan administrative claims			
	Lab test result (gold standard): definition of positive	***N***, patients	Sensitivity	PPV	Kappa	***N***	Sensitivity	PPV	Kappa
**M05**	Any positive RF: ≥ 14 or positive	31,827	0.82 (0.81, 0.82)	0.81 (0.80, 0.82)	0.61 (0.60, 0.62)	1115	0.73 (0.70, 0.76)	0.84 (0.81, 0.87)	0.45 (0.40, 0.50)
	High positive RF: ≥ 42*	24,224	0.86 (0.86, 0.87)	0.73 (0.72, 0.73)	0.61 (0.60, 0.62)	840	0.74 (0.69, 0.78)	0.72 (0.68, 0.76)	0.44 (0.38, 0.50)
	Any positive anti-CCP: ≥ 20 or positive	38,859	0.76 (0.75, 0.76)	0.68 (0.67, 0.69)	0.41 (0.40, 0.42)	261	0.64 (0.56, 0.71)	0.76 (0.68, 0.83)	0.24 (0.13, 0.36)
	High-positive anti-CCP ≥ 60**	34,490	0.82 (0.81, 0.82)	0.63 (0.63, 0.64)	0.45 (0.44, 0.46)	201	0.66 (0.57, 0.75)	0.67 (0.57, 0.76)	0.29 (0.16, 0.42)
	Any positive RF or anti-CCP (RF ≥ 14 or positive) or (anti-CCP ≥ 20 or positive)	43,581	0.76 (0.75, 0.76)	0.82 (0.82, 0.83)	0.51 (0.51, 0.52)	1185	0.73 (0.69, 0.77)	0.84 (0.81, 0.87)	0.45 (0.40, 0.50)
	Any high-positive RF or anti-CCP: RF ≥ 42 or anti-CCP ≥ 60***	40,452	0.83 (0.82, 0.83)	0.71 (0.70, 0.71)	0.53 (0.52, 0.54)	927	0.73 (0.69–0.77)	0.71 (0.67–0.75)	0.42 (0.36, 0.48)
**M06**	Not RF-positive	31,827	0.80 (0.79, 0.80)	0.80 (0.79, 0.81)	0.61 (0.60, 0.62)	1115	0.74 (0.7, 0.79)	0.59 (0.55, 0.64)	0.45 (0.40, 0.50)
	Not RF high-positive*	24,224	0.76 (0.76, 0.77)	0.89 (0.88, 0.89)	0.61 (0.60, 0.62)	840	0.70 (0.65, 0.75)	0.72 (0.67, 0.76)	0.44 (0.38, 0.50)
	Not positive for anti-CCP	38,859	0.66 (0.65, 0.66)	0.74 (0.73, 0.74)	0.41 (0.40, 0.42)	261	0.63 (0.52, 0.73)	0.48 (0.39, 0.57)	0.24 (0.13, 0.36)
	Not high-positive for Anti-CCP**	34,490	0.65 (0.65, 0.66)	0.83 (0.82, 0.83)	0.45 (0.44, 0.46)	201	0.63 (0.52, 0.73)	0.62 (0.52, 0.72)	0.29 (0.16, 0.42)
	Not positive for RF or Anti-CCP-positive	43,581	0.77 (0.76, 0.77)	0.69 (0.68, 0.69)	0.51 (0.51, 0.52)	1185	0.74 (0.70, 0.79)	0.60 (0.56, 0.64)	0.45 (0.40, 0.50)
	Not high-positive for RF or anti-CCP***	40,452	0.71 (0.70, 0.71)	0.83 (0.82, 0.83)	0.53 (0.52, 0.54)	927	0.69 (0.64, 0.73)	0.71 (0.67, 0.76)	0.42 (0.36, 0.48)

*RA* rheumatoid arthritis, *EHR* electronic health record, *PPV* positive predictive value

*Excluded those 14 < RF < 42

**Excluded those 20 < anti-CCP < 60

***Excluded those 14 < <RF < 42 and 20 < anti-CCP < 60

The analysis examining agreement with M05 diagnosis coding according to the recency of rheumatoid factor lab test results were ordered is shown in Table 3. Lab tests ordered on the same day were particularly low (kappa 0.40 in RISE, 0.31 in MarketScan) compared to those where more than 6 months had elapsed between the lab test and the M05 diagnosis code (kappa 0.64 in RISE, 0.51 in MarketScan).

**Table 3:** Agreement between recent rheumatoid factor lab test and M05 diagnosis code according to interval of time since testing occurred

	RISE		MarketScan	
Days between RF lab test and M05 diagnosis code for RA	N	Kappa	N	Kappa
0 (i.e., same day)	1375	0.40 (0.35, 0.45)	81	0.31 (0.11, 0.51)
1–7	691	0.49 (0.42, 0.55)	72	0.44 (0.25, 0.64)
8–30	3850	0.51 (0.48, 0.54)	246	0.46 (0.35, 0.57)
31–180	13,781	0.59 (0.27, 0.60)	847	0.43 (0.37, 0.49)
181–365	6152	0.64 (0.62, 0.66)	267	0.51 (0.41, 0.62)

*RF* rheumatoid factor

**Fig. 1:**
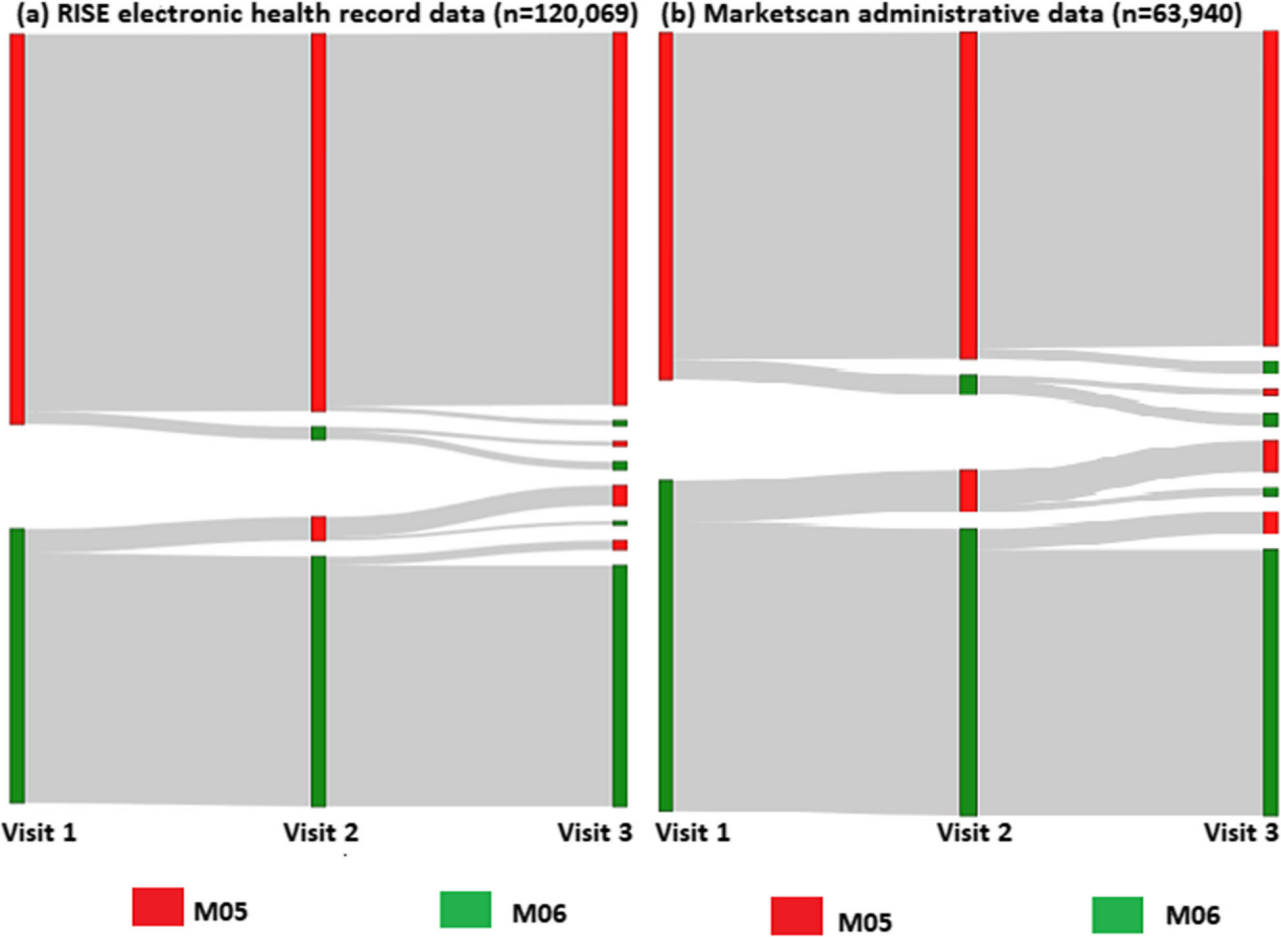
Sankey plot describing transitions from M05 and M06 diagnosis codes across three consecutive RA visits assigned by rheumatologists. **a** RISE electronic health record data (*n* = 120,069). **b** MarketScan administrative claims data (*n* = 63,940)

For the subgroup analysis requiring at least 3 ICD10-coded encounters, 120,069 (RISE) and 63,940 (MarketScan) RA patients qualified for analysis of coding consistency within physician practices. In RISE, 92% of patients were consistently coded by rheumatologists as M05 (56%) or M06 (36%) (Fig. 1a), and only 8% of patients were assigned a mix of M05 and M06 codes. A total of 58.7% (70515/120069) were first diagnosed with M05 (Fig. 1). The parallel results in MarketScan were similar, with 87% of patients always receiving a M05 (47%) or M06 (40%) diagnosis codes, and only 13% of patients shifting. Within the M05 and M06 groups, the specific diagnosis codes assigned to the same patient tended to stay the same. For example, if a patient was first assigned a M06.9 diagnosis (“rheumatoid arthritis, unspecified”), most of these patients that were subsequently assigned any M06 diagnosis code continued to receive M06.9 (87.2% in RISE, 89.4% in MarketScan).

In the analysis examining whether rheumatologists always or never coded for M05/M06 for all their RA patients (Additional file 2), 630 (RISE) and 1950 (MarketScan) rheumatologists contributed information for at least 10 RA patients. The median (IQR) number of patients for each rheumatologist contributing to this analysis was 333 (202, 498) in RISE and 18 (13,29) in MarketScan. The proportion of rheumatologists in RISE who used the M05 or M06 diagnosis codes for either 0% or 100% of their patients was < 2% each. In MarketScan, the proportions were only slightly higher, with 2–6% of rheumatologists always or never using M05 or M06 diagnosis codes for all their RA patients.

Providers seemed to favor a single, specific diagnosis code within each grouping. For example, when any M06 diagnosis code was used by a provider for any of their RA patients, 85.7% of providers in RISE, and 90.8% in MarketScan, used only a single M06 diagnosis code. A few providers used exactly 2 M06 diagnosis codes (10.8% in RISE, 8.4% in MarketScan), and the remainder of providers (< 4% RISE, < 1% MarketScan) ever used three or more specific codes in the M06 group for any of their RA patients. Variability in coding within each group was likewise constrained. For example, in the M06 grouping, the most common diagnosis code used was M06.09 (rheumatoid arthritis without rheumatoid factor, multiple sites), followed by M06.9 (rheumatoid arthritis, unspecified). Together, these two diagnosis codes accounted for more than 80% of all M06 diagnosis codes assigned by providers.

## Discussion

In this real-world evaluation of the validity of the M05 ICD-10 diagnosis codes in two separate data sources to proxy for seropositive RA, we found that M05 had good accuracy (PPV of 81 to 84%) and sensitivity (approximately 73 to 82%) to identify seropositive RA. Performance characteristics were slightly lower for anti-CCP antibody testing, but comparable for the composite of (RF or anti-CCP antibody testing). Requiring additional ICD-10 codes did not meaningfully change the results, as most patients were coded consistently by rheumatologists over the first several visits in the data. These results are likely to be useful to researchers who rely on diagnosis codes to classify patients in real-world data sources in the setting in which lab tests results are not available. Indeed, even in the large U.S. EMR-based RISE rheumatology registry that was used for this analysis covering 2015–2017, approximately 60% of the RA cohort did not have lab test results for either RF or anti-CCP antibody available, presumably because these lab tests were measured at the time of diagnosis and not repeated during the observation period during which RISE data was available.

Prior investigations of coding algorithms to identify RA in large populations using primarily administrative health plan claims data have found that a combination of at least two or more RA diagnosis codes have reasonable validity to correctly classify RA, especially if assigned by a rheumatologists and if the co-occurrence of DMARD use is required [8]. This was the approach that we used to derive the RA cohort. Subsequent classification algorithms to identify RA that incorporated more clinically rich data have investigated the incremental value of adding RF and CCP antibody lab test results. These results could either be directly identified using actual lab values or mention of the results of the lab tests in unstructured physician notes [10, 11]. Algorithms incorporating information about the lab test results were shown to have improved performance compared to administrative data-only algorithms (e.g., PPV = 94% in one single-center study). Subsequent evaluations confirmed the portability of this approach to other institutions [12]. However, these studies were conducted in an era in which ICD-9 coding was used and thus could not directly assess whether ICD-10 diagnosis codes are sufficient by themselves to be used as a surrogate for lab results if actual lab values and unstructured physician notes are not available.

In terms of coding implications, several observations resulting from our analyses may be useful to consider. First, because both RF and anti-CCP antibody testing are typically performed by rheumatologists in the workup of suspected or new-onset RA and results carry prognostic significance [13], the concept of “with rheumatoid factor” embodied by the M05 ICD-10 diagnosis code group may be considered inadequate. Rather, representing the concept of “seropositivity” within ICD-10 may be preferable, which would reflect the presence of either rheumatoid factor and/or anti-CCP antibody. Indeed, a majority of RA patients (typically more than 80%) who are positive for one lab test will be positive for the other. Given the relative infrequency of discordance (i.e., RF+, anti-CCP-; or RF-, anti-CCP+), patients tend to be grouped as seronegative (both negative) or seropositive (either or both positive) in most RA outcome studies [14]. A combined approach also would avoid the need for separately representing the concept of anti-CCP antibody lab test results as part of ICD-10 coding, which avoids added complexity and administrative burden to clinicians assigning these codes.

Strengths of our study include the ability to examine the question of interest in two separate large U.S. scale data sources and the ability to study both EHR data and health plan claim data with diagnoses assigned by rheumatologists. Given that the validity of a clinical RA diagnosis is typically optimal when made by a rheumatologist, we did not examine coding practices or the validity of those codes assigned by non-rheumatologists, which may impact the generalizability of our results in data systems where no information about physician specialty is available. The generalizability of our findings is informed by the evaluation as to whether patients with lab results available were different than those who did not have lab results available. While overall, patients with test results available were relatively similar to those not tested, patients with lab test results available were somewhat younger and less likely to use biologics (in both datasets), consistent with the notion that tested patients likely had shorter disease duration and thus were closer to the time when they had been tested for RF and anti-CCP antibody, as such testing typically occurs at time of diagnosis. However, disease duration was not available in either data sources that we used, and so the coding practices in relation to new onset RA could not be explicitly investigated. Finally, we recognize that although sensitivity and PPVs were good (about 80%), we were unable to explain the reasons why physicians (infrequently) used M05 for seronegative patients or M06 for seropositive patients. We initially anticipated that some EHR systems might automatically map diagnosis codes from ICD-9, yet our focus on the second diagnosis code and inclusion of data through 2017 presumably allowed sufficient time to correct any early miscoding errors occurring after the ICD9 to ICD10 coding transition. We did find that a minority of physicians (up to 6%) seemed to always assign M05 or M06 diagnosis codes for their RA patients (predominantly M06, when this was observed). This could reflect either the actions of an automated EMR-based diagnosis mapping system or the default behavior of the clinician in the setting when lab results were initially unknown and thus an M06 diagnosis code assigned, but where that diagnosis code was never changed to M05 even after the lab results became known.

## Conclusions

In summary, the use of the M05 and M06 ICD10 diagnosis codes appears reasonably useful to identify RA patients with seropositive or seronegative disease, a finding that likely will facilitate clinical research in data systems where lab results are not available. Similar to the fashion in which some EMR vendor systems assign obesity ICD-10 diagnosis codes automatically based on the calculated body mass index, EMR vendors could consider assigning the appropriate M05/M06 RA diagnosis code based on RF and/or anti-CCP lab test results to further improve the accuracy and utility of using structured data (i.e., diagnosis codes) in settings where lab results might not be available.

## Supplementary information

**Additional File 1: Appendix Table 1.** Attrition Table for RISE and Marketscan Data describing RA cohort selection.

**Additional File 2: Appendix Table 2.** Distribution of rheumatologists, grouped by the percentage of RA patient ever given a M05 or M06 diagnosis code.

## Data Availability

All data generated or analyzed during this study are included in this published article and its supplementary information files.

## Abbreviations

ACPA: Anti-citrullinated protein antibody
ACR: American College of Rheumatology
CCP: Anti-cyclic citrullinated peptide
DMARD: Disease-modifying anti-rheumatoid drug
EHR: Electronic health record
ICD: International Classification of Diseases
Kappa: Agreement
M05: Seropositive, rheumatoid factor positive
M06: Seronegative, rheumatoid factor negative
PPV: Positive predicted value
RA: Rheumatoid arthritis
RF: Rheumatoid factor
RISE: Rheumatology Informatics System for Effectiveness
Se: Calculated sensitivity
SMDs: Standardized mean differences
