# Supplementary material for: Use of ICD-10 diagnosis codes to identify seropositive and seronegative rheumatoid arthritis when lab results are not available
Source: Arthritis Res Ther. 2020 Oct 15;22:242. doi: 10.1186/s13075-020-02310-z (PMC7560310; doi:10.1186/s13075-020-02310-z)
Supplement: Supplementary file 1 — Additional File 1: Appendix Table 1. Attrition Table for RISE and Marketscan Data describing RA cohort selection. [file 13075_2020_2310_MOESM1_ESM.docx]

**Appendix Table 1: Attrition Table for RISE and Marketscan Data describing RA cohort selection**

|  | RISE | Marketscan |
| --- | --- | --- |
| ICD-10 diagnosis for RA from rheumatologist, Q4 2015 - Q4 2017 | 188,487 | 119,670 |
| Second ICD-10 RA diagnosis, separated from first by >= 7 and <=365 days | 154,594 | 89,918 |
| At least 1 prescription or administration of a DMARD (conventional, biologic, or targeted synthetic) [main study population, Table 1] | 134,406 | 78,787 |
| With at least three rheumatologist visits [Figure 1, subgroup analysis] | 120,069 | 63,940 |

DMARDs: disease modifying anti-rheumatic drug; RA: rheumatoid arthritis
