# Supplementary material for: Use of ICD-10 diagnosis codes to identify seropositive and seronegative rheumatoid arthritis when lab results are not available
Source: Arthritis Res Ther. 2020 Oct 15;22:242. doi: 10.1186/s13075-020-02310-z (PMC7560310; doi:10.1186/s13075-020-02310-z)
Supplement: Supplementary file 2 — Additional File 2: Appendix Table 2. Distribution of rheumatologists, grouped by the percentage of RA patient ever given a M05 or M06 diagnosis code. [file 13075_2020_2310_MOESM2_ESM.docx]

**Appendix Table 2: Distribution of rheumatologists, grouped by the percentage of RA patient ever given a M05 or M06 diagnosis code**

|  | RISE (N=630) | | MarketScan (N=1,950) | |
| --- | --- | --- | --- | --- |
| Proportion of RA patients in each physician’s practice assigned an M05 or M06 diagnosis, % | M05 (Seropositive) | M06 (Seronegative) | M05 (Seropositive) | M06 (Seronegative) |
| 0 | 5 (0.79) | 0 (0.00) | 72 (3.69) | 40 (2.05) |
| 0, <25 | 43 (6.83) | 79 (12.54) | 149 (7.64) | 287 (14.72) |
| >=25, <50 | 102 (16.19) | 308 (48.89) | 346 (17.74) | 694 (35.59) |
| >=50, <75 | 312 (49.52) | 163 (25.87) | 792 (40.62) | 544 (27.90) |
| >=75, <100 | 167 (26.51) | 72 (11.43) | 515 (26.41) | 273 (14.00) |
| 100 | 1 (0.16) | 8 (1.27) | 76 (3.90) | 112 (5.74) |

Note: the M05 and M06 diagnosis codes were ascertained as ever occurring and not time-varying
